# Phylogenetic Relationships and Potential Functional Attributes of the Genus *Parapedobacter*: A Member of Family *Sphingobacteriaceae*

**DOI:** 10.3389/fmicb.2020.01725

**Published:** 2020-09-04

**Authors:** Shekhar Nagar, Chandni Talwar, Shazia Haider, Akshita Puri, Kalaiarasan Ponnusamy, Madhuri Gupta, Utkarsh Sood, Abhay Bajaj, Rup Lal, Roshan Kumar

**Affiliations:** ^1^Department of Zoology, University of Delhi, Delhi, India; ^2^Department of Biotechnology, Jaypee Institute of Information Technology, Noida, India; ^3^P.G.T.D, Zoology, R.T.M Nagpur University, Nagpur, India; ^4^School of Biotechnology, Jawaharlal Nehru University, New Delhi, India; ^5^The Energy and Resources Institute, New Delhi, India; ^6^Environmental Biotechnology and Genomics Division, CSIR-National Environmental Engineering Research Institute, Nagpur, India; ^7^P.G. Department of Zoology, Magadh University, Bodh Gaya, India

**Keywords:** *Parapedobacter*, Bat operon, pectinases, inositol, ortholog analysis

## Abstract

The genus *Parapedobacter* was established to describe a novel genus within the family *Sphingobacteriaceae* and derives its name from *Pedobacter*, with which it is shown to be evolutionarily related. Despite this, *Parapedobacter* and *Pedobacter* do not share very high 16S rRNA gene sequence similarities. Therefore, we hypothesized whether these substantial differences at the 16S rRNA gene level depict the true phylogeny or that these genomes have actually diverged. Thus, we performed genomic analysis of the four available genomes of *Parapedobacter* to better understand their phylogenomic position within family *Sphingobacteriaceae*. Our results demonstrated that *Parapedobacter* is more closely related to species of *Olivibacter*, as opposed to the genus *Pedobacter*. Further, we identified a significant class of enzymes called pectinases with potential industrial applications within the genomes of *Parapedobacter luteus* DSM 22899^T^ and *Parapedobacter composti* DSM 22900^T^. These enzymes, specifically pectinesterases and pectate lyases, are presumed to have largely different catalytic activities based on very low sequence similarities to already known enzymes and thus may be exploited for industrial applications. We also determined the complete *Bacteroides* aerotolerance (Bat) operon (*batA, batB, batC, batD, batE*, hypothetical protein, *moxR*, and *pa3071*) within the genome of *Parapedobacter indicus* RK1^T^. This expands the definition of genus *Parapedobacter* to containing members that are able to tolerate oxygen stress using encoded oxidative stress responsive systems. By conducting a signal propagation network analysis, we determined that BatD, BatE, and hypothetical proteins are the major controlling hubs that drive the expression of Bat operon. As a key metabolic difference, we also annotated the complete *iol* operon within the *P*. *indicus* RK1^T^ genome for utilization of all three stereoisomers of inositol, namely myo-inositol, scyllo-inositol, and 1D-chiro-inositol, which are abundant sources of organic phosphate found in soils. The results suggest that the genus *Parapedobacter* holds promising applications owing to its environmentally relevant genomic adaptations, which may be exploited in the future.

## Introduction

The genus *Parapedobacter* was first described by Kim et al. (2007), under the family *Sphingobacteriaceae* of the phylum Bacteroidetes. Based on the highest gene sequence identity (<90%), *Parapedobacter* was found to be akin to the genus *Pedobacter* (Kim et al., 2007). Currently, nine validly published species that have been isolated from a wide range of ecological habitats represent this genus (www.bacterio.net/genus/parapedobacter). These are *Parapedobacter koreensis* (dried rice straw) (Kim et al., 2007), *Parapedobacter soli* (ginseng field soil) (Kim et al., 2008), *Parapedobacter luteus* and *Parapedobacter composti* (cotton waste compost) (Kim et al., 2010), *Parapedobacter pyrenivorans* (Zhao et al., 2013) and *Parapedobacter indicus* (polluted soils) (Kumar et al., 2015), *Parapedobacter deserti* (plant stem) (Liu et al., 2017), *Parapedobacter lycopersici* (rhizospheric soil) (Kim et al., 2017), and *Parapedobacter defluvii* (sewage treatment plant) (Yang et al., 2017). Altogether, the genomic understanding of this genus has been lacking since its identification more than a decade ago (Kim et al., 2007). Here, we uncover the evolutionary relationships and define the functional attributes of this genus through four available genome sequences: *P*. *indicus* RK1^T^ [hexachlorocyclohexane (HCH) dumpsite), *P*. *koreensis* Jip14^T^ (dried rice straw), *P*. *composti* DSM 22900^T^, and *P*. *luteus* DSM 22899^T^ (cotton waste compost).

Our study provides a view of the phylogenomic relatedness of *Parapedobacter* with other genera of family *Sphingobacteriaceae*. To address this, we adopted an alignment-free method for computing pairwise dissimilarity values among the genomes that correspond to the number of substitution events separating two leaves over the evolutionary course (Criscuolo, 2019). These methods are becoming increasingly popular to address the challenges in computational requirements, runtime, and need for manual interventions with large genomic datasets, as in this study. We found *Parapedobacter* to be more closely related to the genus *Olivibacter* than to *Pedobacter*, which was previously reported based on 16S rRNA gene sequence similarities (Kim et al., 2007; Kumar et al., 2015). Through pangenome analyses, we clarified the genomic factors that shaped the unique distribution of *Parapedobacter* spp. while identifying functions that distinguished each species through their unique genomic contents. With the aim of finding the ecological relevance of *Parapedobacter* spp., we determined an industrially significant class of enzymes called pectinases encoded within the genomes of *Parapedobacter* spp. The pectinases identified within the genomes of *Parapedobacter* spp. shared low sequence similarity with the other known enzymes of this class and are likely to have important industrial applications. Our study also revealed the microaerophilic nature of one of the species, *P. indicus* RK1, as determined from the presence of the *Bacteroides* aerotolerance (Bat) operon genes within its genome and the expression of these genes under aerobic stress. This further widens the genomic definitions of the genus *Parapedobacter*, which was previously suggested to harbor strictly aerobic members (Kim et al., 2007). Through a systems biology approach, we conducted a signal propagation analysis of proteins encoded by the Bat operon genes to report the key proteins in their communication. We also report differences in the metabolic preferences of *Parapedobacter* spp., more specifically in the utilization of three stereo-isomers of inositol. Overall, the study resolved the peculiarities at the phylogenomic level as well as at the functional level in members of this novel genus.

## Materials and Methods

### Genome Sequencing and Assembly, Annotations and Genomic Features

The genome of *P. indicus* RK1 was sequenced by the Joint Genome Institute, CA, USA, under the Genomic Encyclopedia of Type Strains, Phase III (KMG-III), using the Illumina HiSeq 2500-1TB platform, and assembled using the SOAPdenovo assembler (Li et al., 2010). The sequence data for *P. indicus* RK1 have been submitted to the National Center for Biotechnology Information (NCBI) Genome database (GenBank: FOQO00000000). Subsequently, three other available genomes were obtained for comparative analyses: *P. koreensis* Jip14 (GenBank: FNZR00000000), *P. composti* DSM 22900 (GenBank: FOLL00000000), and *P. luteus* DSM 22899 (GenBank: FUYS00000000). The annotations were performed using the Rapid Annotation using Subsystems Technology (RAST) (Aziz et al., 2008) server with gene caller Glimmer-3 (Delcher et al., 2007). The presence of clustered regularly interspaced short palindromic repeats (CRISPR) elements was analyzed using the CRISPRFinder server (Grissa et al., 2007). The degree of genome completeness was predicted by analyzing 107 essential copy genes using hidden Markov models (Dupont et al., 2012).

### Phylogenomic Analysis

The genome-based phylogeny of validly published members of family *Sphingobacteriaceae* (with available genomes on or before May 21, 2020) was inferred using an alignment-free distance-based method with JolyTree (Criscuolo, 2019). Briefly, for each pair of genomes, the pairwise *p*-distance (i.e., the proportion of aligned nucleotide differences) value was calculated using Mash v.2.1 (Ondov et al., 2016), followed by the correction of each value into a numerical quantity that is proportional to the evolutionary distance between the compared genomes. Further, the phylogeny was constructed using the balanced minimum-evolution (BME) method (Desper and Gascuel, 2002) based on an improved neighbor-joining principle using FastME v.2.1.5.1 (Lefort et al., 2015). Finally, the branch confidence values of the inferred tree were calculated using the REQ program (Guénoche and Garreta, 2001), which estimates the rate of elementary quartets (REQ) for each branch of the tree from the associated distance matrix. The program thus assigns confidence values from 0 to 1, where higher values denote branches fully supported by the pairwise evolutionary distances. To further decipher the genomic heterogeneity, the genome-wide average nucleotide identity (ANI) trend was explored using an ANIb (BLAST based) approach (Konstantinidis and Tiedje, 2005) and the heatmap was plotted using the pheatmap package in R (Kolde and Kolde, 2015).

### Pangenome Analyses

The pangenome of *Parapedobacter* was studied by means of the anvi'o workflow (Eren et al., 2015). Proteins were predicted within each genome using Prodigal (Hyatt et al., 2010). Each protein in every genome was compared with every other protein using the NCBI BLASTP program (–*use-ncbi-blast*). The sequence similarity and algorithm sensitivity were estimated using bitscore and MCL inflation (van Dongen and Abreu-Goodger, 2012) parameters while identifying clusters in a protein similarity search. The program was run at –*min-bit* = 0.5, which denotes the sequences to be at least 50% identical in length for clustering together, and –*min-inflation* was set to 10 for maximum sensitivity (from a scale of 2–10). The genomes were subsequently clustered based on their core and accessory gene clusters using the Euclidean distance (Gower, 1982) and Ward linkage (Ward, 1963). The genes were annotated for Clusters of Orthologous Groups (COG) functional classes implemented in the anvi'o pangenomics workflow (Eren et al., 2015). The strain-specific or unique gene clusters of each genome were distributed into different functional COG categories and a heatmap was generated using the pheatmap package in R (Kolde and Kolde, 2015). Further, the core genome was also estimated using the GET_HOMOLOGUES pipeline using an 80% cutoff value for both query coverage (QC) and percentage identity (PI).

### Comparative Functional Analysis and Genome Orthology

For each genome, proteins were annotated to be placed in COG categories by using RPS-Blast against the NCBI COG database (Tatusov et al., 2001) and a heatmap was plotted using average clustering. The genomes were searched for similar gene contents using the orthoMCL algorithm that is available in GET_HOMOLOGUES (Contreras-Moreira and Vinuesa, 2013). As the genomes were highly variable, we determined their core contents using QC and percentage identity at regular intervals of 5%, starting from 100% and going down to 50% (for both parameters), to classify the protein families that are widely shared among the genomes. The shared sequences within each interval were subsequently annotated into COG categories using RPS-Blast against the NCBI COG database (Tatusov et al., 2001). Further, the amino acid sequences of each genome were searched against Pfam (Finn et al., 2016) and the profiles of unique and shared protein families among the genomes were compared. The protein sequences of pectinesterases and pectate lyases annotated as unique Pfam families in *P*. *luteus* DSM 22899 and *P*. *composti* DSM 22900 were compared by using the BLASTP program on the NCBI database based on PI and QC with the top 10 hits. Annotations of genes involved in inositol utilization and those coding for pectate lyase and pectinesterase identified by Glimmer 3 in RAST were checked manually on UniProt (Pundir et al., 2016).

### Computational Systems Biology Analysis of Bat Proteins

To date, the protein–protein interaction (PPIs) studies of *Parapedobacter* spp. have not been performed and thus no PPI data for this genus are available. We followed the systems biology approach to analyze the proteins of the Bat operon cluster (*n* = 8) to identify the importance of each of these proteins. For this, the eight protein (BatA, BatB, BatC, BatD, BatE, hypothetical protein, MoxR, and PA3071) sequences of *P. indicus* RK1 were searched against those of the closest phylogenetic neighbor, *Pedobacter heparinus* (Steyn et al., 1998), using BLASTP to find homologous proteins. PPI data for *P. heparinus* were retrieved from the STRING database v.10 (Szklarczyk et al., 2015), which consists of known and predicted PPIs including direct (physical) and indirect (functional) interactions among sets of proteins. The interactions with a confidence score equal to or >0.4 (default parameters) were considered. The PPI networks were visualized in Cytoscape v.3.5 (Shannon et al., 2003) and analyzed using the Network analyzer to find the important protein. Further, to explore the signal communications of these important proteins in the cell, we carried out signal propagation analysis. For this, each protein was separately analyzed for direct interaction with other proteins, which form the shells of nodes at a distance *r*. These interacting partners (*r* = 1) were searched in the STRING database and network growth was constructed up to *r* = 8 while taking care of the repeated proteins. Perl script v.5.18.2.2 was used to construct the propagation of network growth.

The average degree of nodes in the shell at distance *r* was calculated as $\frac{{<k}^{2}>}{<k>}$, and the average residual degree as $\frac{\left({<k}^{2}>-<k>\right)}{{<k}^{2}>}$. So, the probability that the nodes at a distance *r* interlink with the nodes in the shell, |D (*r*)|, is given by $|D\;\left(r\right)|=\frac{{<k}^{2}>}{{<k}^{2}>-<k>}e^{\propto r}$, where $\alpha =\frac{{<k}^{2}>}{<k>}$ is the rate of network expansion (Barzel and Barabási, 2013).

### *Bacteroides* Aerotolerance (Bat) Gene Expression in *Parapedobacter indicus* RK1

*P*. *indicus* RK1, previously reported to be isolated from HCH-contaminated soil by our laboratory (Kumar et al., 2015), was grown to mid-exponential phase under aerobic as well as under microaerophilic conditions (CO_2_ incubator containing 5% CO_2_) at 30°C in Luria–Bertani medium. Growth was monitored at regular intervals by measuring OD values at 600 nm by means of a spectrophotometer. Primers for the eight Bat operon genes, namely *batA, batB, batC, batD, batE, pa3071*, hypothetical protein, and *moxR*, were designed (Supplementary File S1) and the genes were amplified to confirm their presence within the genome. The amplified products were cleaned using a NucleoSpin Gel and PCR Clean-up Kit according to the manufacturer's instructions. The eluted products were further Sanger sequenced and reconstructed using Sequencing Analysis v.5.1.1 (Applied Biosystems) and confirmed as Bat operon genes through BLASTn analysis. The expression of these genes under aerobic as well as microaerophilic conditions was studied by harvesting the cells after 24 h of growth under these conditions. Total RNA was isolated using an RNeasy Mini Kit (Qiagen) according to the manufacturer's instructions. Complementary DNA (cDNA) was synthesized by using a Revert Aid First Strand cDNA synthesis kit (Thermo Scientific) according to the manufacturer's instructions. The expression of Bat operon genes was confirmed through amplification using cDNA.

## Results and Discussion

### General Genomic Attributes and Distribution of *Parapedobacter* Across the Phylogenetic Clade

In this study, we analyzed four draft genomes of genus *Parapedobacter*; namely, *P. indicus* RK1, *P. koreensis* Jip14, *P. luteus* DSM 22899, and *P. composti* DSM 22900. As they belong to a novel genus, so far no genomic studies have been reported. The genome size and %G+C content of these *Parapedobacter* strains varied between 4.6–6.1 Mbp and 48–50%, respectively (Supplementary Table 1). These features can be attributed to their evolution in different ecological settings, as the complexity of the environment differs with their respective habitats. For example, strain RK1 was isolated from an HCH dumpsite (Kumar et al., 2015) and its genome was found to harbor genes for aromatic compound degradation pathways, such as the beta-ketoadipate pathway (muconate cyclo-isomerase, succinyl-CoA:3-ketoacid-coenzyme A transferase subunit A and B, and mandelate racemase) and salicylate and gentisate catabolism (salicylate esterase and fumarylacetoacetate hydrolase) (Sharma et al., 2015; Talwar et al., 2020). Further, the abundance of membrane transport genes (*n* = 208) and genes related to the stress response contributing toward osmotic stress (*n* = 54) and oxidative stress (*n* = 51) were significantly abundant within the genome of strain RK1. The abundance of these genes has been suggested as an adaptation in other organisms under toxic soil environments and might contribute toward the adaptability of *P. indicus* RK1 in an HCH-contaminated environment (Sangwan et al., 2012; Talwar et al., 2020).

**Figure 1:**
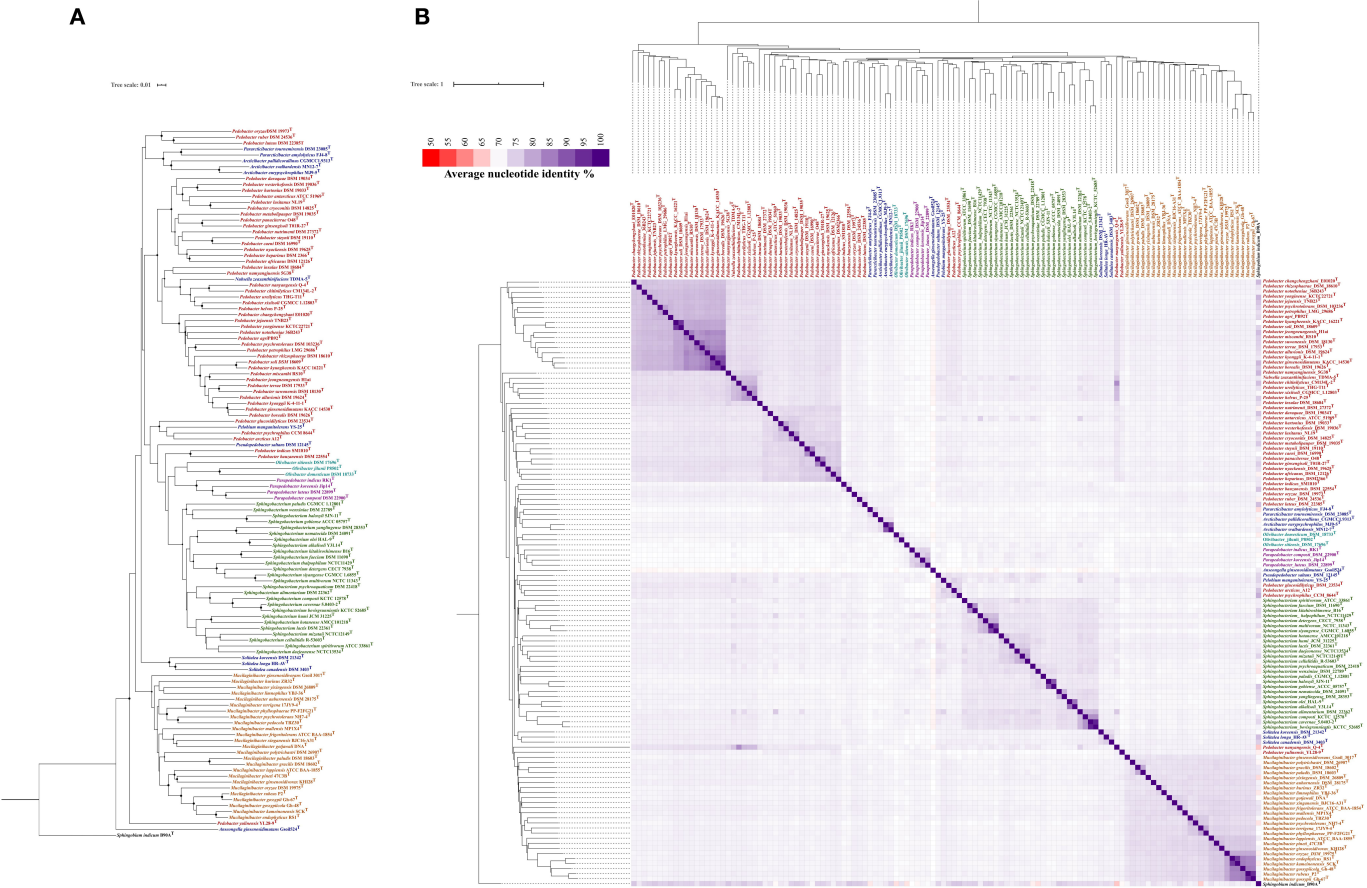
Phylogenetic analysis of genus *Parapedobacter*. **(A)** Phylogenetic tree constructed from the pairwise distance estimates from unaligned genomes of the validly published species of family *Sphingobacteriaceae. Sphingobium indicum* B90A was used as an outgroup. The tree was constructed in JolyTree (Criscuolo, 2019) using the balanced minimum-evolution method, based on an improved Neighbor Joining principle using FastME v.2.1.5.1 (Lefort et al., 2015). The branch confidence values were computed using the REQ program (Guénoche and Garreta, 2001) and values in the range 0.7–1 are shown with circles of corresponding sizes on branches of the inferred tree. **(B)** Phylogeny reconstructed from BLAST-based pairwise average nucleotide identity (ANIb) using available genomes of validly published species of the family *Sphingobacteriaceae* and *Sphingobium indicum* B90A as an outgroup. The pairwise %ANIb values among genomes were compared using the Manhattan distance and hierarchical clustering and a heatmap was constructed.

Additionally, we aimed to study the evolutionary relationships of *Parapedobacter* spp. among other genera of the family *Sphingobacteriaceae*. For this, we carried out parallel whole genome-based phylogeny assessments of all validly published species of the family *Sphingobacteriaceae* using pairwise genomic dissimilarity measures based on substitution events and BLAST-based whole-genome average nucleotide identities (ANIb) analyses. The former method performs alignment-free, pairwise whole-genome comparisons and has been used previously for resolving phylogenies from large genomic datasets (Parks et al., 2018; Badell et al., 2020; Tarlachkov et al., 2020). Although the genus *Parapedobacter* derives its name from *Pedobacter* based on 16S rRNA gene sequence similarity (Kim et al., 2007), the inferred trees from both the described methods revealed a close association of *Parapedobacter* with the genus *Olivibacter* (Figures 1A,B). This provides evidence for the greater significance of whole genome-based evaluation of the phylogeny over a single 16S rRNA gene-based approach in bacterial taxonomy (Mahato et al., 2017). However, the clustering of *Parapedobacter* with *Olivibacter* was supported by low confidence scores (Figure 1A). Thus, sequencing and addition of more genomes of *Olivibacter* spp. would reveal their phylogenetic association with *Parapedobacter* more clearly. In addition, ANIb trends also revealed the *Parapedobacter* spp. to be related to *Arcticibacter* and *Pararcticibacter*; however, additional genomes of recognized species will unveil the genomic relatedness of these genera more clearly (Figure 1B). Within the genus *Parapedobacter*, a whole genome-based dissimilarity matrix clustered *P*. *composti* DSM 22900 and *P*. *luteus* DSM 22899 together and *P*. *koreensis* Jip14 and *P. indicus* RK1 joined the clade distantly. These results were consistent with the 16S rRNA gene sequence analysis based on which *P. indicus* RK1 was reported to be closest to *P*. *koreensis* Jip14 (Kumar et al., 2015). *P*. *composti* DSM 22900 and *P*. *luteus* DSM 22899 are isolates from same habitat, i.e., cotton waste compost, which may account for their high evolutionary relatedness inferred based on the substitution events. However, ANIb-based phylogeny placed *P*. *luteus* DSM 22899 with *P*. *koreensis* Jip14 (ANI = 79.08%), while *P*. *luteus* DSM 22899 and *P*. *composti* DSM 22900 shared 76.3% ANIb. The pairwise similarities between all *Parapedobacter* genomes ranged between 73.9 and 79.0%, clearly separating them from other members of the family *Sphingobacteriaceae* (Figure 1B). Therefore, although the *Parapedobacter* were originally identified and named based on their 16S rRNA gene sequence relatedness with *Pedobacter* spp., sequencing and addition of whole genomes in the family *Sphingobacteriaceae* has resolved their taxonomic position with other genera in the family.

### Pangenomic Attributes of *Parapedobacter*

**Figure 2:**
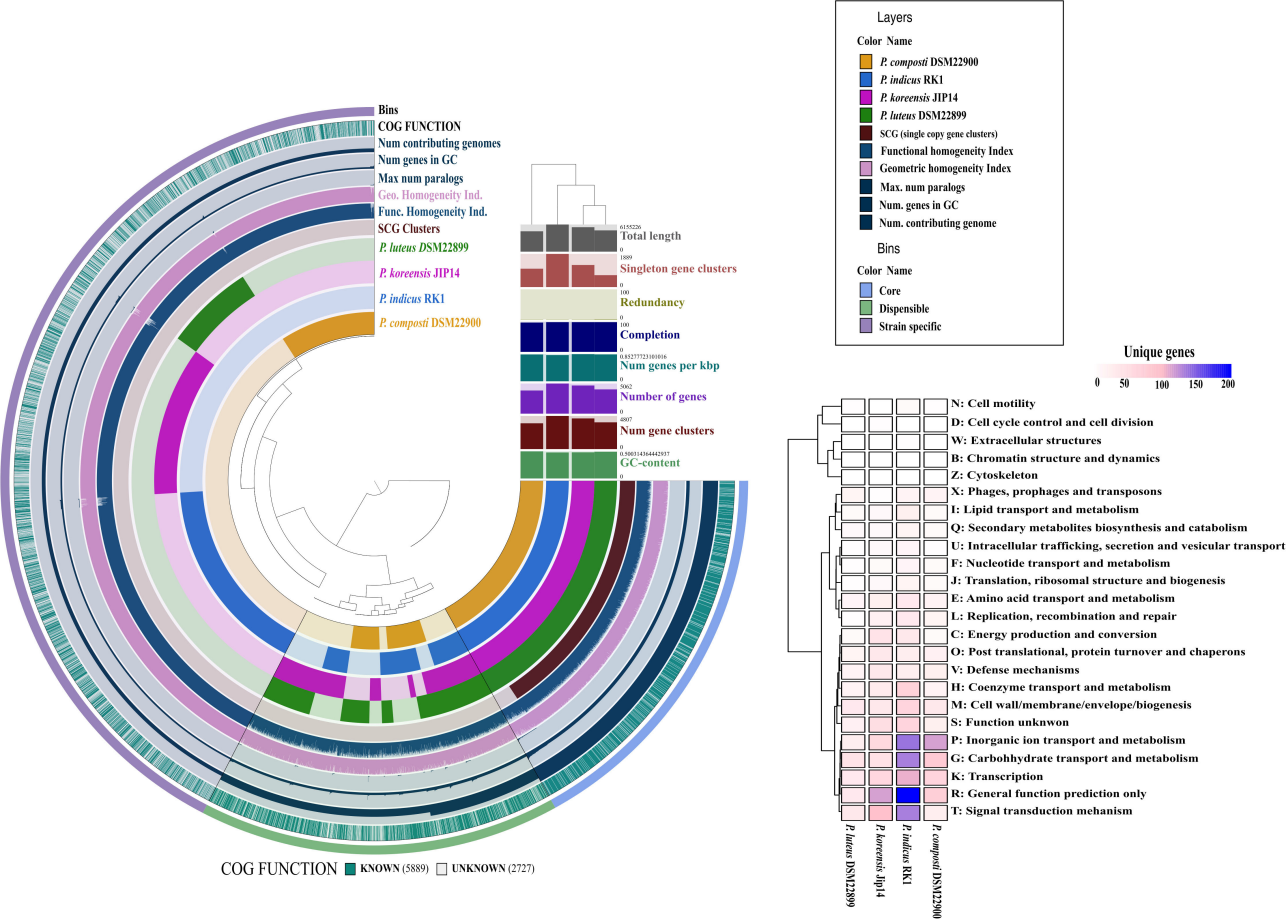
Comparative circular phylogram of *Parapedobacter* genomes based on common genetic attributes and gene frequencies. The genomes are arranged in radial layers with their genomic contents organized into core and accessory gene contents computed using the Euclidean distance and the Ward linkage method. The clustering of genomes is shown as a dendrogram based on core and accessory genomic contents at the top right corner of the circular phylogram. The strain-specific contents of each of the genomes were annotated into different COG functional categories and plotted as a heatmap, shown on the right.

The genomes were more than 98% complete, as estimated from the presence of essential single-copy genes (Dupont et al., 2012; Kumar et al., 2017) (Supplementary Table 1). This removed bias from the results of whole-genome comparisons based on core and accessory gene contents from subsequent analyses. The pangenome distributed into 17,761 gene clusters (minbit = 0.5; min-inflation = 10; distance: Euclidean; linkage: Ward). Further, the core genome analysis revealed a total of 1,084 genes present in four *Parapedobacter* spp. genomes (QC = 80% and PI = 80%). The genomes were also revealed to be highly non-redundant (98.6–99.3%) (Figure 2). This reflects that the *Parapedobacter* spp. have a tendency to reduce genomic paralogy yet they maintain functional diversity, which might have implications in niche-specific adaptations (Mendonça et al., 2011).

More than one-fourth (28.0%) of the pangenome was formed from the strain-specific gene contents for which high abundance was noted in the COG classes: general function prediction (R), inorganic ion transport and metabolism (P), carbohydrate transport and metabolism (G), signal transduction mechanisms (T), and transcription (K). Thus, genomic fractions that greatly distinguished strain-specific contents from core contents were assigned to inorganic ion transport and metabolism (P) and signal transduction mechanisms (T). Specifically, these functions were abundant in the strain-specific contents of the *P*. *indicus* RK1, *P*. *composti* DSM 22900, and *P*. *koreensis* Jip14 genomes. Being an isolate from HCH-polluted soil, the abundance of unique genes involved in signal transduction and inorganic ion transport and metabolism within *P*. *indicus* RK1 might enable the organism to sense environmental signals and use chemotaxis; this is consistent with the previous assertions in species of different genera isolated from this stressed niche (Verma et al., 2014; Sharma et al., 2015; Talwar et al., 2020). *P*. *indicus* RK1 harbored the highest unique gene content (10.9%) followed by *P*. *koreensis* Jip-14 (7.4%), *P*. *composti* DSM 22900 (6.1%), and *P*. *luteus* DSM 22899 (3.9%) (Figure 2). Thus, the large strain-specific content might account for the largest of the *Parapedobacter* genomes, represented by *P*. *indicus* RK1 at 6.2 Mbp. Since, *P*. *composti* DSM 22900 and *P*. *luteus* DSM 22899 both inhabited the same habitat of cotton waste compost, it was interesting to outline the major functional differences among them that placed them at a greater distance at the pangenomic level. *P*. *composti* DSM 22900 harbored 20.1% of its unique gene content involved in inorganic ion transport and metabolism, compared with that in *P*. *luteus* DSM 22899 (7.9%) (Figure 2). The unique contents of the two genomes were also highly diverged for functions of transcription (K), carbohydrate transport and metabolism (G), and general function prediction only (R), which were all greatly abundant (more than 2-fold) in *P*. *composti* DSM 22900 (Figure 2). Thus, although evolving in the same ecological niche, the two bacterial species might have adapted differently with respect to their key functions, as has been described previously (de la Haba et al., 2019). Since strain RK1 has large strain-specific genomic attributes that may be phage induced, as has already been documented for many rapidly evolving bacterial genomes (Lopez et al., 2012; Blesa et al., 2018), we wanted to look for CRISPR elements. While genomes of all three strains except *P. composti* DSM 22900 harbored CRISPR elements (Supplementary Table 1), only one CRISPR from *P*. *koreensis* Jip14 showed significant similarity to *Caldilinea aerophila* DSM 14535 (QC = 76%, PI = 75%, *E* = 2e-14). It was found to code for gliding motility-associated C-terminal domain-containing protein, suggesting the phage-mediated acquisition of this protein in the strain. CRISPRs from the two other genomes, *P*. *indicus* RK1 and *P*. *luteus* DSM 22899, were not found to code for any known protein. The low number of CRISPRs detected within *Parapedobacter* spp. suggested their non-frequent encounters with the phages. To uncover the important functions of the genus, we further analyzed *Parapedobacter* genomes to compare them based on broad COG functional categories and further delved into key functions under these COG categories.

### Comparative Account of Potential Functional Attributes

**Figure 3:**
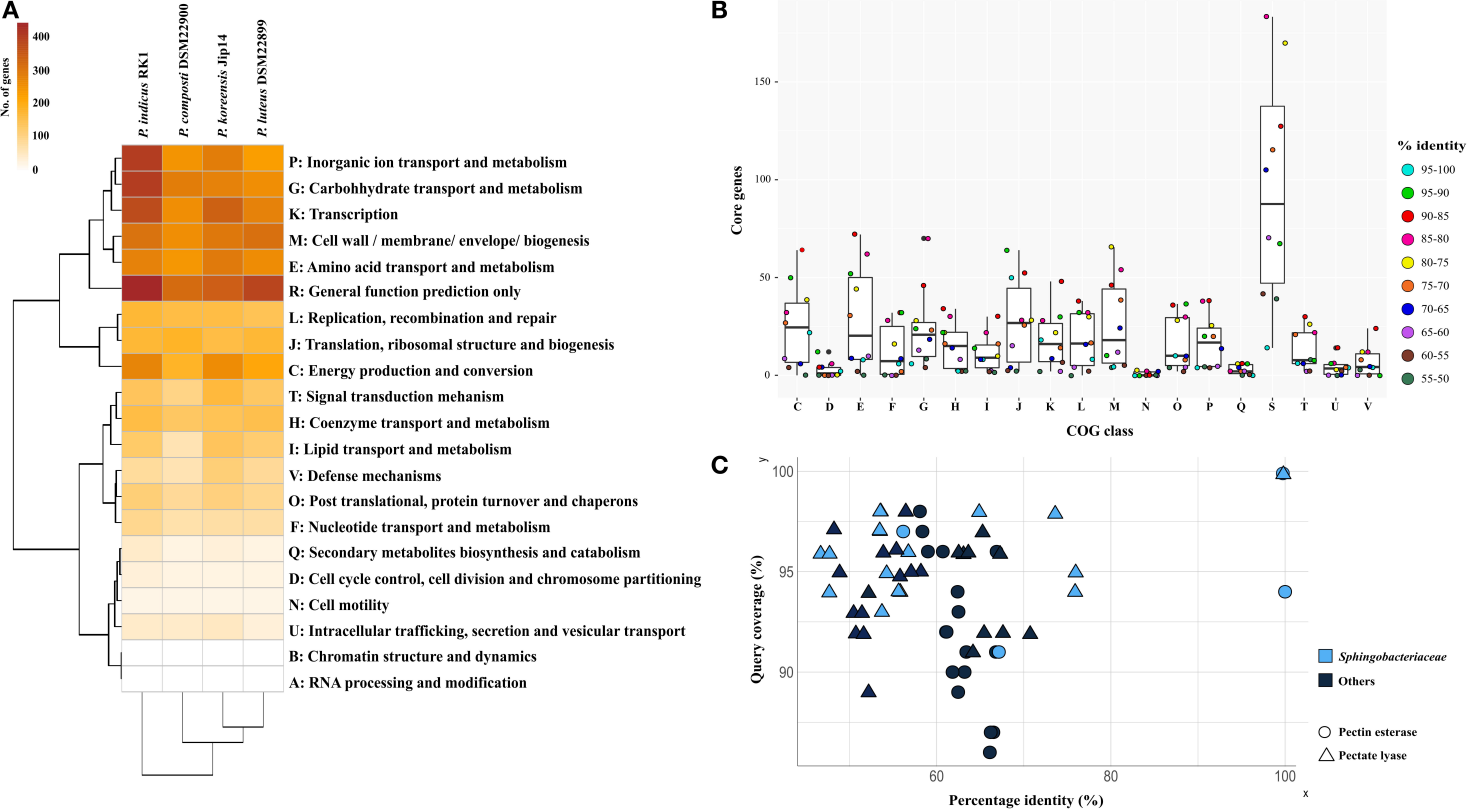
Comparative functional analysis of genus *Parapedobacter*. **(A)** Annotation of proteins using RPS-Blast against the COG database. Pearson correlation and complete clustering were used to generate the heatmap. **(B)** The distribution of proteins shared at discrete intervals of percentage identity and coverage cutoff in different COG categories showing the relative similarities of proteins under each functional category. **(C)** Top 10 hits of pectinesterase and pectate lyase proteins identified within genomes of strains DSM 22899 and DSM 22900 showing sequence identities plotted as a function of percentage identity (*x*-axis) vs. the coverage cutoff (*y*-axis). The shape and color of each protein denote the protein type and whether the protein belongs to the family *Sphingobacteriaceae*, respectively.

Based on whole-genome-encoded functions, *P*. *indicus* RK1 separated out from the rest of the genomes as opposed to core- and accessory genome-based clustering (Figure 3A). The strain had an abundance of genes involved in unknown functions, carbohydrate transport and metabolism, and transcription. In addition, inorganic ion transport and metabolism was highest in *P*. *indicus* RK1, which was also revealed from strain-specific contents analysis as discussed above. Thus, the strain possessed mechanisms for the utilization of resources as well as for adapting to environmental pressures imposed by HCH pollution.

To identify the level of COG functional similarity among the genomes, we further determined the core genes using both QC and PI at intervals of 5%, starting from 100 to 95% and going down to 55 to 50%, and annotated these genes from the genomes to reveal the relative abundance of COG functions at these discrete windows (Figure 3B). This was done with aim of identifying the percentage similarity between core functional genes to provide an estimate of the functional relatedness of *Parapedobacter* genomes. The maximum gene content of each of these genomes was found to be shared at above 55% identity and QC. The annotations revealed that the majority of these core functional genes shared highest similarity in the range 90% to 85% under each COG category, with the exceptions of translation, ribosomal structure, and biogenesis (J; 95–90%) and cell wall/membrane/envelope biogenesis (M; 80–75%) (Figure 3B). This revealed the high similarity of the functional core genes of *Parapedobacter* genomes.

We observed *P*. *luteus* DSM 22899 and *P*. *composti* DSM 22900 to be least abundant for all Pfam categories compared with the two other species (data not shown). This can be justified as these are comparatively smaller among the four members in terms of coding potential (Figure 2). In spite of having the smaller coding potential, these strains possessed unique protein families under carbohydrate transport and metabolism. Most importantly, the strains possessed pectate lyase (pfam09492) and pectinesterase (pfam01095) as unique enzymes. Since, they form an important class of industrial enzymes, we were interested in studying their diversity within *Parapedobacter*. In DSM 22899, two variants of pectinesterases and one pectate lyase were annotated while strain DSM 22900 maintained one and three copies of these genes, respectively. This suggested the important functional significance of these enzymes in the two strains in the context of their habitat of cotton waste compost. Pectin is one of the major polysaccharides in plant cell walls. It is modified and degraded by a group of naturally occurring pectinases that are essential for cell wall extension and the recycling of plant materials (Voragen et al., 2009). Pectinesterase breaks down pectin into pectate and methanol (Fries et al., 2007), while pectate lyase catalyzes the eliminative cleavage of de-esterified pectin and thus mediates cell wall degradation and fruit softening (Marín-Rodríguez et al., 2002). Pectate lyases of plant-associated bacteria enable them to utilize the pectin from dead or living plants as a carbon source for growth (Hugouvieux-Cotte-Pattat et al., 2014). As strains DSM 22899 and DSM 22900 are isolates from cotton waste composts used for the cultivation of the oyster mushroom (*Pleurotus ostreatus*) (Kim et al., 2010), they might be assumed to naturally help in bio-scouring of cotton fiber using pectinases encoded within their genomes. To determine whether this is an acquired phenotype in these strains, we compared the percentage identities of each of these proteins with the top 10 hits obtained through BLASTP on NCBI (Supplementary File S2). The analysis revealed that the two copies of pectinesterase annotated within the genome of DSM 22899 were 100% identical at 94% QC while these proteins were only 66.8% similar to those of DSM 22900 (91% QC), displaying the differences between them despite being isolated from the same habitat (Figure 3C). Moreover, the two pectinesterase proteins of DSM 22899 shared similarity in the range of 63–66% with their top 10 neighbors, none of which belonged to the family *Sphingobacteriaceae*. The topmost hit of pectinesterase protein of DSM 22900 after DSM 22899 was that from *Pedobacter glucosidilyticus* (56.1% PI at 97% QC), which belongs to the family *Sphingobacteriaceae*. Percentage identities with all other hits ranged between 59 and 62% at low QCs with sequences from genera not belonging to the family *Sphingobacteriaceae* (Figure 3C). Similarly, pectate lyase proteins from the two strains were also checked for their closest hits. The only copy of pectate lyase within DSM 22899 showed similarity to that of DSM 22900 (76% PI; 95% QC). It showed similarities in the range 48–53% to the rest of its neighbors, most of which were not from *Sphingobacteriaceae* (Figure 3C). Out of the three copies of pectate lyase present in DSM 22900, one showed maximum similarity to proteins of genus *Sphingobacterium* (55.98% PI; 94% QC); however, the two other copies showed maximum similarity to proteins from *Catalinimonas* (67.22% PI; 96% QC) and *Rufibacter* (56.49% PI; 98% QC) (Figure 3C; Supplementary File S2). Pectinases find several other industrial applications, including clarification and stabilization of fruit juices; softening of pickles; fermentation of coffee, cocoa, and tea; preparation of jams and jellies; as supplements in animal feed for optimal absorption of nutrients; for increasing bioethanol production; and in paper and wine manufacturing (Kubra et al., 2018). Microbial pectinases are preferred over other chemical methods as they are ecofriendly and energy efficient (Maldonado and Strasser de Saad, 1998; Kashyap et al., 2001; Garg et al., 2016). Thus, these enzymes from DSM 22899 and DSM 22900 can be further explored for their industrial implications.

We further annotated the complete cluster of Bat operon genes within the genome of *P. indicus* RK1, which were lacking in the three other genomes. The Bat proteins were first identified within the genome of *Bacteroides fragilis* to have a role in aerotolerance (Tang et al., 1999) and have been reported to be involved in the bacterial response to oxidative stress in other species (Meuric et al., 2008). Despite their identification more than two decades ago, the literature on Bat proteins has been very limited. Through this study, we performed genome-based analysis of these proteins for the first time by analyzing the signal propagation in their PPI network using a systems biology approach. We further studied the expression of these genes qualitatively under oxidative stress to understand this responsive mechanism.

### Analysis of Signal Propagation in Protein–Protein Interactions of Bat Proteins and Their Expression Under Microaerophilic Stress

**Figure 4:**
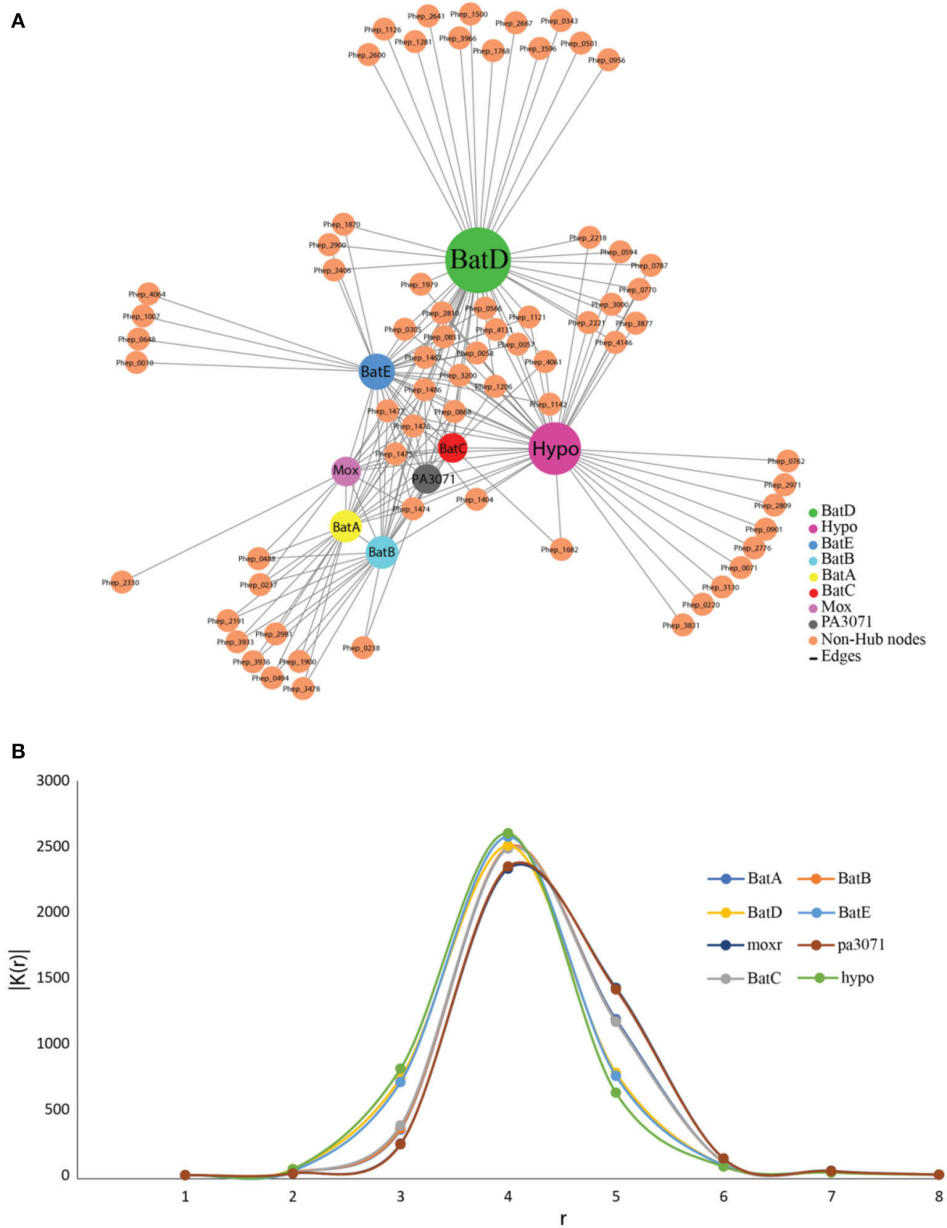
The protein–protein interaction network and signal propagation of Bat operon genes of *P. indicus* RK1. **(A)** Expanded view of the network imported from Cytoscape, where nodes represent proteins and edges represent their physical interactions. All the nodes and edges are in orange and gray, respectively. The existence of the few sparsely distributed main gene hubs, namely BatD, hypothetical protein, BatE, BatB, BatA, BatC, MoxR, and PA3071, in the network is indicated by a color other than orange. **(B)** Propagation of signal in the protein–protein interaction network growth of BatD, Hypo, BatE, BatB, BatA, BatC, MoxR, and PA3071 is highlighted by colored lines.

PPIs of Bat proteins of *P*. *indicus* RK1 were studied through those of its closest neighbor, *P. heparinus*, with available datasets on the STRING database (Figure 4). The genome was used as a reference following a preliminary BLASTP analysis of Bat proteins of *P*. *indicus* RK1 (BatA, BatB, BatC, BatD, BatE, hypothetical protein, MoxR, and PA3071), which revealed those from *P. heparinus* as the topmost hits. The network comprised 78 proteins involved in 216 interactions, as visualized in Cytoscape v.3.5. The network analysis identified highly interacting proteins (hubs) among these eight proteins (Figure 4A). BatD showed maximum interaction with degree 55 followed by hypothetical protein (Hypo; degree: 53), BatE (degree: 39), BatB (degree: 30), BatA (degree: 29), BatC (degree: 23), MoxR (degree: 22), and PA3071 (degree: 21). These results suggested that BatD is the most important protein of the Bat operon. In order to understand the expression of the other proteins, we carried out computational signal propagation, which could suggest the communication of these proteins in *P*. *indicus* RK1. The propagation of a signal from a specific node in a large network to other nodes at a distance *r* was measured using the probability that the nodes at a distance *r* link with the specific node, |D (*r*)|. This parameter indicates how perturbation at a certain node affects the stability in the network and its topological characteristics. We calculated |D (*r*)| for each of the proteins (BatA, BatB, BatC, BatD, BatE, hypothetical protein, MoxR, and PA3071) (Figure 4B), and found that |D (*r*)| first increased exponentially with *r*. This indicated that the signal processing from the respective hubs was active (propagation of perturbation from the respective hubs increased) and efficient. Then this propagation of signal was found to saturate at a particular value of *r*, denoted by *r*_*s*_. The saturation value of *r*_*s*_ for all eight proteins was *r*_*s*_ = 4. The peak value of |D (*r*)| was maximum for BatD, BatE, and hypothetical protein when *r*_*s*_ < 4, whereas MoxR and PA3071 had maximum propagation when *r*_*s*_ > 4. Thus, the |D (*r*)| decreased as a function of *r* owing to the contraction of the shells of nodes, which allowed a decrease in signal propagation. The analysis indicated that BatD, BatE, and hypothetical protein had the maximum impact on the regulatory network of aerotolerance, and the remaining hubs (BatB, BatC, and BatA) had similar controlling capability (Figure 4B).

The presence of genes associated with the Bat operon was confirmed within the genome of *P*. *indicus* RK1 through the amplification of the eight genes (BatA, BatB, BatC, BatD, BatE, hypothetical protein, MoxR, and PA3071) using custom-designed primers for each gene (Supplementary Figure S1; Supplementary File S1). The expression of these Bat genes in strain RK1 was analyzed by growing the culture under aerobic as well as microaerophilic conditions. All eight Bat-associated genes could be amplified from the cDNA obtained from cells of *P*. *indicus* RK1 following 24 h of growth under aerobic and microaerophilic conditions (data shown for microaerophilic conditions; Supplementary Figure S1). This supports our hypothesis based on comparative genomic analysis that the genus might harbor microaerophilic species that have not yet been identified. The study provides preliminary insights based on qualitative data and further studies focused independently on the nature of growth of *Parapedobacter* spp. under different oxic conditions would reveal the quantitative expression of each of these Bat operon-associated genes and their underlying mechanisms in *Parapedobacter indicus* RK1, which is beyond the scope of this genome-based study. However, to gain more genome-based insights, we further performed comparative genomic analysis of *Parapedobacter* spp. for oxidative stress response-related genes and proteins.

### Comparative Genomic Basis of the Oxidative Stress Response in *Parapedobacter* spp

One of the key classes of enzymes that are inherently associated with growth under different oxic conditions in bacteria are the ribonucleotidereductases (RNR), which catalyze the synthesis of deoxyribonucleotide triphosphates (dNTPs). RNRs of classes Ib (*nrdE, nrdF, nrdI*) and class III (*nrdD, nrdG*), which are reported to be active under aerobic and anaerobic conditions, respectively (Jordan and Reichard, 1998), were annotated within the genome of RK1 (Supplementary Table 2). BLASTN analysis of *nrdEF* genes encoding class Ib RNR and cofactor assembly protein *nrdI* showed the highest similarities to proteins of *P*. *koreensis* Jip14 (Supplementary Table 2). The genes of anaerobic class III RNR *nrdDG* also showed the highest similarity to the proteins of *P*. *koreensis* Jip14. RNRs are categorized as class I, II, or III depending upon radical generation, cofactor requirement, and oxygen dependence (Jordan and Reichard, 1998). Class I represents aerobic enzymes generating a tyrosyl radical with an iron–oxygen center and class II functions under both aerobic and anaerobic conditions generating adenosylcobalamin. Class III are anaerobic enzymes generating a glycyl radical from *S*-adenosylmethionine and an iron–sulfur cluster. Class I RNRs are further divided into class Ia, Ib, and Ic, depending upon the metal center required for protein radical generation (Jordan and Reichard, 1998). The presence of both aerobic and anaerobic classes of RNRs in *P*. *indicus* RK1 strengthened the prediction for its survival at different oxic conditions.

Further, we studied the presence/absence patterns of five *osr* elements, which are expressed during oxidative stress in bacteria (Smalley et al., 2002; Supplementary Table 3). Three genes – namely, class Ia RNR-encoding gene *nrdA*; an aspartate decarboxylase-encoding gene, *asdA;* and a putative outer-membrane protein similar to *susC* of *Bacteroides thetaiotaomicron* – which are known to be highly induced during periods of oxidative stress in bacteria were annotated within all *Parapedobacter* spp. (Smalley et al., 2002). The two other genes – namely, cation efflux pump-encoding gene *czcD* and a heat shock protein, *htpG* – were absent in all the strains. To note, Smalley et al. (2002) reported that these two genes were not as highly induced under oxidative stress. In addition, several other genes coding for enzymes participating in the oxidative stress response were annotated within *Parapedobacter* genomes, including manganese superoxide dismutase, alkyl hydroperoxide reductase (*ahp*), rubredoxin, catalase, glutathione peroxidase, and non-specific DNA binding proteins which are responsible for scavenging superoxide radicals and peroxides, thereby combating oxidative stress (Rocha and Smith, 1999; Fu et al., 2007; Hagelueken et al., 2007). Thus, the annotation of these oxidative stress response systems hinted toward possible aerotolerance mechanisms used by *Parapedobacter* spp. for protection from cellular reactive oxygen species.

We were further interested in the comparative metabolic profiles of the *Parapedobacter* spp. Since the comparative functional profiling of genomes revealed the abundance of carbohydrate metabolism genes within the *P*. *indicus* RK1 genome, we looked into specific functional genes that were enriched under this category. As one of the distinguishing metabolic characteristics, *P*. *indicus* RK1 was found to harbor a complete gene cluster for degradation of all three stereo-isomers of inositol, namely myo-inositol, scyllo-inositol and 1D-chiro-inositol, which were studied in detail.

### Inositol Utilization in *P*. *indicus* RK1

**Figure 5:**
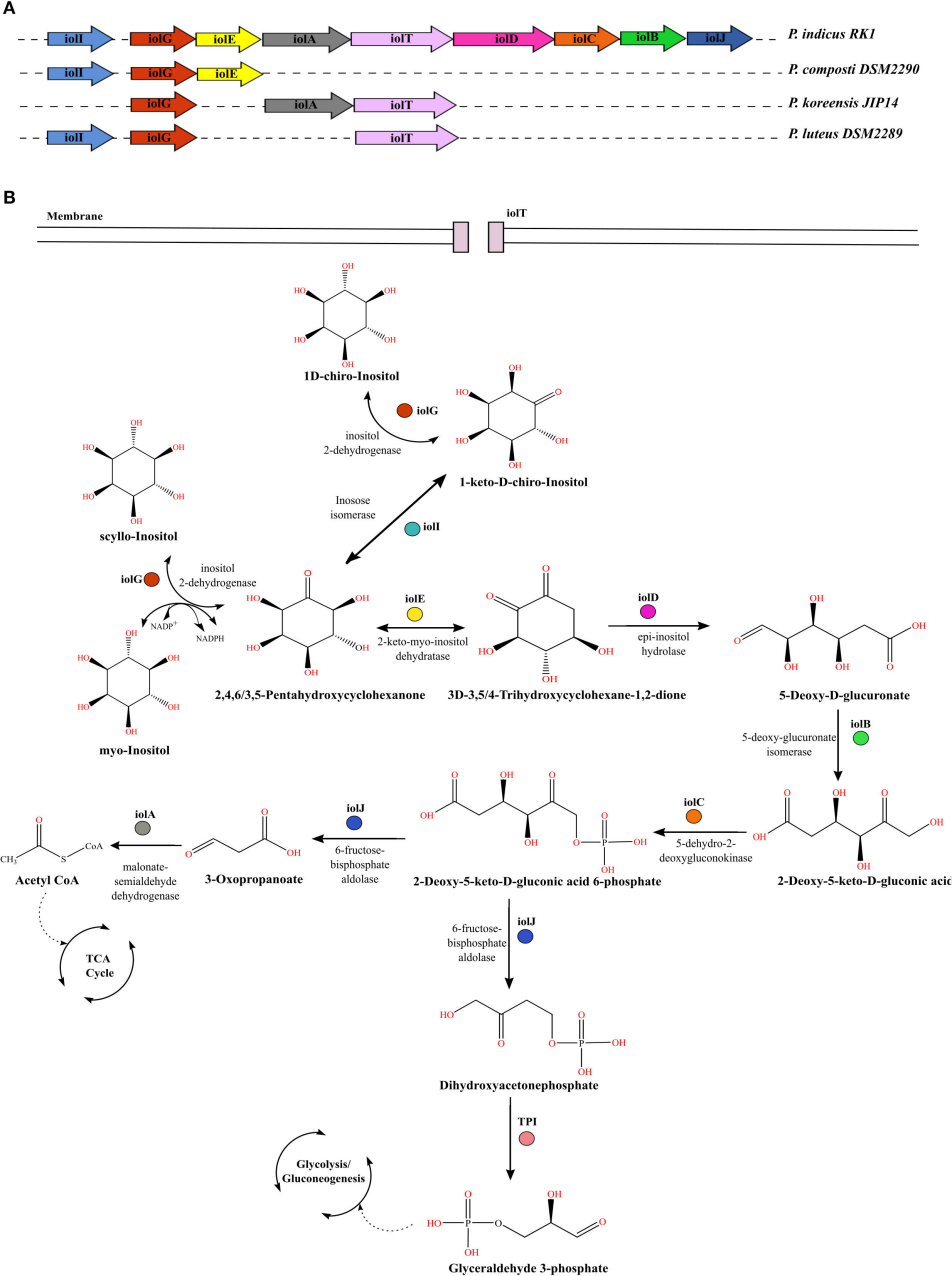
Comparative genomic profiles for utilization of inositol stereo-isomers by *Parapedobacter* spp. **(A)** Organization of *iol* genes in the operon within the genome of strain RK1 as compared with other strains that lack the complete *iol* gene cluster. **(B)** Pathway for catabolism of three stereo-isomers of inositol, namely myo-inositol, scyllo-inositol, and chiro-inositol, in bacteria. Each gene encoding the enzyme responsible for catalyzing a reaction is denoted by the same color as in panel **(A)**.

The carbohydrate myo-inositol commonly exists as the mono- or phosphorylated form (commonly known as phytic acid) and corresponds to more than 80% of organic phosphate in the soil (Turner et al., 2002). Many soil microbes utilize these forms as an energy source. However, the utilization differs among organisms based on the genetic organization of these gene clusters. The molecular genetics of inositol metabolism is best studied in *Bacillus subtilis*, which is shown to metabolize all three stereo-isomers using the *iol* operon (Yoshida et al., 1997; Yamaoka et al., 2011). We annotated the complete *iol* operon only in strain RK1, which is an isolate of HCH-contaminated soil, as the other strains lacked its complete gene cluster (Figure 5A). For uptake of myo-inositol from the extracellular environment strain RK1 possessed a sodium/myo-inositol co-transporter *iolT*. Upon entering into the bacterial cell, myo-inositol is converted to intermediates that enter glycolysis and subsequently the tricarboxylic acid cycle in successive reactions catalyzed by enzymes encoded by the *iol* operon genes (Yoshida et al., 1997; Yamaoka et al., 2011) (Figure 5B). Inside the bacterial cell, inositol-2-dehydrogenase encoded by *iolG* initiates the catabolism of inositol isomers. The strain RK1 harbored two variants of this gene, *iolG* and *iolG1*. The presence of *iolI*, which encodes an inosose isomerase, suggested the potential interconversion of scyllo-inositol into 1D-chiro-inositol via an intermediate, namely 1-keto-D-chiro-inositol. The successive steps in the catabolism of inositol are catalyzed by the enzymes inosose dehydratase (*iolE*), epi-inositol hydrolase (*iolD*), 5-deoxy-glucuronate isomerase (*iolB*), 5-dehydro-2-deoxygluconokinase (*iolC*), fructose-bisphosphate aldolase (*iolJ*), and malonate-semialdehyde dehydrogenase (*iolA*), as illustrated in Figure 5B (Krings et al., 2006). The catabolic products glyceraldehyde-3-phosphate and acetyl coenzyme A then enter the glycolytic and tricarboxylic acid cycle, respectively, for energy production. In addition to the presence of a complete gene cluster for inositol catabolism, a large copy number of *iolG* (*n* = 23) and the presence of variant form *iolG1* within the genome of strain RK1 suggest extensive utilization of inositol in this organism as an acquired mechanism for utilization of myo-inositol reserves in soil.

## Conclusion

The present study defines the evolutionary relationships of the genus *Parapedobacter* with members of the family *Sphingobacteriaceae*. Further, this comparative study uncovered the microaerobic nature of *P*. *indicus* RK1, which harbors the Bat operon as well as other genes, such as the *osr* and *rnr* group, that participate in the response to oxidative stress. The expression of Bat operon-associated genes in *P*. *indicus* RK1 was confirmed when grown under aerobic and microaerophilic conditions. Further, for the first time using a systems biology approach, signal propagation analysis of the proteins encoded by the Bat operon genes was performed. The results suggest that BatD, BatE, and hypothetical protein had the maximum impact on the regulatory network of aerotolerance and thus these are the major players of the Bat operon. The study also identified industrially important enzymes of the classes pectinesterases and pectate lyases within two bacterial strains, namely *P*. *composti* DSM 22900 and *P*. *luteus* DSM 22899. This opens up new avenues to study their enzymatic activity both *in vitro* and *in vivo* for their use as industrial enzymes in the future.

## Data Availability Statement

All datasets presented in this study are included in the article/Supplementary Material.

## Author Contributions

SN, RL, AB, and RK planned the study. SN, RK, CT, SH, AP, KP, MG, US, and AB performed the experiments and analysis. SN, CT, SH, AP, and RK wrote the manuscript. RL and RK critically reviewed the manuscript and improved it. All authors read and approved the final manuscript.

## Conflict of Interest

The authors declare that the research was conducted in the absence of any commercial or financial relationships that could be construed as a potential conflict of interest.

## Supplementary Material

Supplementary Figure S1 Electrophoretogram (0.8% agarose gel matrix) of Bat operon genes of *Parapedobacter indicus* RK1. **(A1,A2)** Lane 1, Gene ruler (Fermentas no. SM0331, USA); lanes 2–7 in **(A1)** and lanes 2–3 in **(A2)**, amplified Bat operon genes from strain RK1. Sizes (in bp) of the different genes are labeled on each band. **(B)** Bat operon genes amplified from cDNA of strain RK1 following 24 h of microaerophilic growth.
