# Supplementary figures and images for: Phylogenetic Relationships and Potential Functional Attributes of the Genus Parapedobacter: A Member of Family Sphingobacteriaceae
Source: Front Microbiol. 2020 Sep 4;11:1725. doi: 10.3389/fmicb.2020.01725 (PMC7500135; doi:10.3389/fmicb.2020.01725)

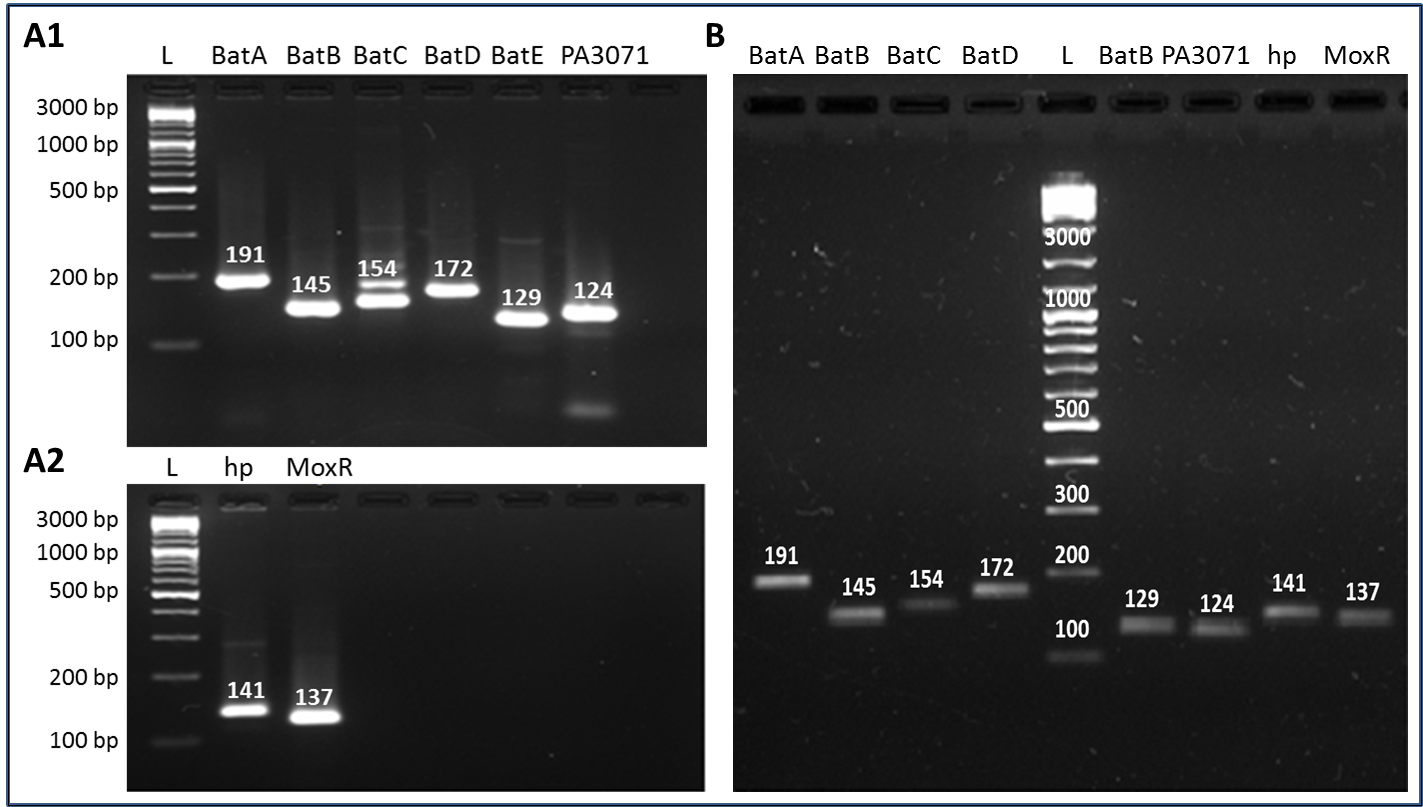

Supplement: Supplementary Figure S1 — Electrophoretogram (0.8% agarose gel matrix) of Bat operon genes of Parapedobacter indicus RK1. (A1,A2) Lane 1, Gene ruler (Fermentas no. SM0331, USA); lanes 2–7 in (A1) and lanes 2–3 in (A2), amplified Bat operon genes from strain RK1. Sizes (in bp) of the different genes are labeled on each band. (B) Bat operon genes amplified from cDNA of strain RK1 following 24 h of microaerophilic growth. [file Image_1.png]
