# Supplementary material for: Phylogenetic Relationships and Potential Functional Attributes of the Genus Parapedobacter: A Member of Family Sphingobacteriaceae
Source: Front Microbiol. 2020 Sep 4;11:1725. doi: 10.3389/fmicb.2020.01725 (PMC7500135; doi:10.3389/fmicb.2020.01725)
Supplement: Supplementary file 2 [file Data_Sheet_1.docx]

**Bat A primer**

|  | **Sequence (5'->3')** | **Template strand** | **Length** | **Start** | **Stop** | **Tm** | **GC%** | **Self complementarity** | **Self 3' complementarity** |
| --- | --- | --- | --- | --- | --- | --- | --- | --- | --- |
| **Forward primer** | GGTGGTATTCAGCGGGGAAA | Plus | 20 | 408 | 427 | 60.03 | 55.00 | 4.00 | 2.00 |
| **Reverse primer** | CGGTGAGCAGAATCACCACT | Minus | 20 | 598 | 579 | 60.04 | 55.00 | 5.00 | 1.00 |
| **Product length** | 191 | | | | | | | | |

**Bat B primer**

|  | **Sequence (5'->3')** | **Template strand** | **Length** | **Start** | **Stop** | **Tm** | **GC%** | **Self complementarity** | **Self 3' complementarity** |
| --- | --- | --- | --- | --- | --- | --- | --- | --- | --- |
| **Forward primer** | TCCTCAACCACATCAGCACC | Plus | 20 | 443 | 462 | 59.96 | 55.00 | 2.00 | 0.00 |
| **Reverse primer** | TCTTCGTGGTTCTCGCCATC | Minus | 20 | 587 | 568 | 60.11 | 55.00 | 3.00 | 0.00 |
| **Product length** | 145 | | | | | | | | |

**Bat C primer**

|  | **Sequence (5'->3')** | **Template strand** | **Length** | **Start** | **Stop** | **Tm** | **GC%** | **Self complementarity** | **Self 3' complementarity** |
| --- | --- | --- | --- | --- | --- | --- | --- | --- | --- |
| **Forward primer** | CAGGACAACAAAGACCCGGA | Plus | 20 | 532 | 551 | 59.89 | 55.00 | 4.00 | 2.00 |
| **Reverse primer** | CTTCCAATAGACGCTCGGCA | Minus | 20 | 685 | 666 | 60.18 | 55.00 | 3.00 | 1.00 |
| **Product length** | 154 | | | | | | | | |

**Bat D primer**

|  | **Sequence (5'->3')** | **Template strand** | **Length** | **Start** | **Stop** | **Tm** | **GC%** | **Self complementarity** | **Self 3' complementarity** |
| --- | --- | --- | --- | --- | --- | --- | --- | --- | --- |
| **Forward primer** | ACCCCTTCGAACGGTTCTTC | Plus | 20 | 773 | 792 | 59.97 | 55.00 | 8.00 | 1.00 |
| **Reverse primer** | TTCAGTTCCGTGCGGTCTAC | Minus | 20 | 944 | 925 | 60.04 | 55.00 | 4.00 | 2.00 |
| **Product length** | 172 | | | | | | | | |

**Bat E primer**

|  | **Sequence (5'->3')** | **Template strand** | **Length** | **Start** | **Stop** | **Tm** | **GC%** | **Self complementarity** | **Self 3' complementarity** |
| --- | --- | --- | --- | --- | --- | --- | --- | --- | --- |
| **Forward primer** | ACCTATACGCATGGCAACCC | Plus | 20 | 434 | 453 | 60.18 | 55.00 | 4.00 | 0.00 |
| **Reverse primer** | CGCCATTGTGATCAACCTGC | Minus | 20 | 562 | 543 | 60.18 | 55.00 | 6.00 | 2.00 |
| **Product length** | 129 | | | | | | | | |

**hp PA3071 primer**

|  | **Sequence (5'->3')** | **Template strand** | **Length** | **Start** | **Stop** | **Tm** | **GC%** | **Self complementarity** | **Self 3' complementarity** |
| --- | --- | --- | --- | --- | --- | --- | --- | --- | --- |
| **Forward primer** | AGAGGAGCGTGAAATGACCG | Plus | 20 | 216 | 235 | 60.11 | 55.00 | 2.00 | 2.00 |
| **Reverse primer** | GGCTGAAAACGCAAGTACCG | Minus | 20 | 339 | 320 | 60.11 | 55.00 | 4.00 | 2.00 |
| **Product length** | 124 | | | | | | | | |

**hp primer**

|  | **Sequence (5'->3')** | **Template strand** | **Length** | **Start** | **Stop** | **Tm** | **GC%** | **Self complementarity** | **Self 3' complementarity** |
| --- | --- | --- | --- | --- | --- | --- | --- | --- | --- |
| **Forward primer** | GCTGGCTCTGGGAAAATTGC | Plus | 20 | 567 | 586 | 60.11 | 55.00 | 4.00 | 3.00 |
| **Reverse primer** | GTCTGTTCCATCGCCGGTAT | Minus | 20 | 707 | 688 | 59.90 | 55.00 | 4.00 | 2.00 |
| **Product length** | 141 | | | | | | | | |

**Mox-R protein**

|  | **Sequence (5'->3')** | **Template strand** | **Length** | **Start** | **Stop** | **Tm** | **GC%** | **Self complementarity** | **Self 3' complementarity** |
| --- | --- | --- | --- | --- | --- | --- | --- | --- | --- |
| **Forward primer** | CGATCAGACGTTTCCGTTGC | Plus | 20 | 462 | 481 | 59.91 | 55.00 | 4.00 | 2.00 |
| **Reverse primer** | TTTTGGGGTAGCCGATCACC | Minus | 20 | 598 | 579 | 60.03 | 55.00 | 4.00 | 3.00 |
| **Product length** | 137 | | | | | | | | |
